# Comparative Analysis of Thermophysical Properties of Functional Epoxy Matrix Composites Reinforced with Glass or Carbon Fibers in the Context of Heat Transfer Anisotropy

**DOI:** 10.3390/ma18214838

**Published:** 2025-10-22

**Authors:** Andrzej J. Panas, Zbigniew Leciejewski, Judyta Sienkiewicz, Mirosław Nowakowski

**Affiliations:** 1 Faculty of Mechatronics, Armament and Aerospace, Military University of Technology, 2 Kaliskiego Str., 00-908 Warsaw, Poland; zbigniew.leciejewski@wat.edu.pl; 2 Air Force Institute of Technology, 6 Księcia Bolesława Str., 01-494 Warsaw, Poland

**Keywords:** carbon-fiber reinforced polymer, glass-fiber reinforced polymer, thermophysical properties, comparative studies

## Abstract

The paper presents comprehensive and complementary studies of the thermophysical properties of functional composite structures. The term functional in this case means the study of the structure while maintaining its post-production imperfections, as opposed to the study of material samples prepared solely for this purpose. The paper presents the results of experimental studies, followed by an analysis of thermophysical properties characterizing heat accumulation and anisotropic heat transfer of two types of utility composites. Composites with an epoxy matrix and two types of reinforcement, glass and carbon fibers, were studied. The research program included micro- and macrostructural analysis and comprehensive thermogravimetric, microcalorimetric and thermal diffusivity measurements. In the studies of heat transfer phenomena, the directional dependence of properties was considered. Attention was focused on maintaining high temperature resolution of measurements, and the effect of repeated temperature exposure was also determined. The results of the research are the determined quantitative and qualitative characteristics, including the temperature dependence of a set of thermophysical properties of the tested materials. Key findings include higher thermal stability and a significant thermal anisotropy ratio in the graphite-reinforced polymer composite compared to the glass-reinforced polymer composite, which exhibited a lower onset decomposition temperature. The results offer crucial data for engineering calculations, structural analyses, and defining operational limits. Analysis of the results provides insight into possible design and operational problems of real structures in relation to model data.

## 1. Introduction

Composites are materials composed of two or more distinct phases, combined to achieve superior properties compared to the individual components [1]. Structural composites vary widely depending on the type, size, and form of the reinforcement and the manufacturing method, and they can be tailored to meet demanding requirements such as high strength, thermal stability, or resistance to cyclic thermal loads.

Carbon- and glass-fiber reinforced polymer composites, due to their low thermal expansion, high strength at elevated temperatures, and favorable strength-to-weight ratios, are widely used in aerospace, military, and civilian applications, including engine nozzles, combustion chambers, and brake discs [2,3,4]. Their thermophysical properties, strongly influenced by microstructure (density, porosity, and anisotropy), are critical but remain insufficiently reported.

Challenges include the anisotropy of properties, which complicates test sample preparation, uniform testing procedures, and result interpretation. Epoxy matrices, in turn, limit the upper temperature range and increase susceptibility to environmental effects, leading to property changes over time [5]. Therefore, studies should consider thermal history, repeated thermal cycles, and complementary methods to ensure reliable results [6]. Despite advances in manufacturing, ensuring repeatability in composite structures remains difficult, making comprehensive and carefully designed investigations essential.

Recent studies emphasize that the thermophysical and microscopic properties of carbon-fiber reinforced epoxy composites (CFRPs) and glass-fiber reinforced epoxy composites (GFRPs) strongly influence their performance in service environments. The defect initiation and growth, such as matrix microcracking, fiber–matrix debonding, and void expansion, are critical factors governing durability and thermal stability. For instance, fatigue-related studies confirm that microstructural defects significantly affect lifetime and property degradation under cyclic thermal and mechanical loads [7]. Moreover, in [8] it was proved that interlaminar shear strength decreases under both water and alkaline exposure, but the mechanisms differ—water primarily causes physical swelling and debonding, while alkaline solution induces chemical attack on the glass fibers. Therefore, understanding the evolution of thermophysical as well as mechanical properties in relation to microstructural changes under varying service conditions remains essential for reliable material design and qualification.

This research investigates the thermophysical properties of industrial-grade composite structures, which contain natural structural imperfections. The research focused on glass fiber-reinforced polymer (GFRP) and carbon fiber-reinforced polymer (CFRP) composites. Thermophysical properties were examined beyond classic thermodynamic parameters, to include thermomechanical characteristics such as viscoelasticity [9,10,11,12].

The purpose of the work was to characterize phase transformations and material behavior under thermal loading, with a focus on heat accumulation, heat transfer, and anisotropy. To achieve this, we employed a dedicated, author-developed temperature variation program, previously described in the literature [6,13], which ensures reliable results in both the heating and cooling modes of the microcalorimeter.

The results aim to provide data on real-world structures that may differ from model data for “perfect” structures, while also highlighting how the measurement procedure can influence the final results.

## 2. Materials and Methods

This research utilized three types of composite materials:

- two glass-fiber reinforced polymer composites (3A Composites Mobility S.A., Mielec, Poland) with different thicknesses:
  ○ with a thickness of 6.2–6.4 mm-designated GFRP A;
  ○ with a thickness of 4.5–5.2 mm-designated GFRP B;
- a carbon-fiber reinforced polymer composite (Rega Yacht sp. z o.o., Ropczyce, Poland) reinforced with carbon fabric layers (designated “CFRP”).

The research described in this publication is part of a broader, comprehensive research program aimed at determining the actual, effective thermophysical and thermomechanical properties. The work encompasses both practical and theoretical aspects. The practical part concerns the provision of appropriate structural and design data. From a theoretical perspective, both the potential impact of material imperfections and the research methodology within individual test methods are important. In this case, the focus is on thermal properties: heat transport and storage.

Initially, the tested composites were subjected to microstructural analysis. Imaging techniques included a KEYENCE VHX-6000 digital microscope (KEYENCE Corporation, Osaka, Japan) and a Phenom ProX scanning electron microscope (Phenom-World B.V., Eindhoven, The Netherlands). Microscopic images were analyzed to determine individual layer thicknesses, porosity levels, and the volume fractions of constituent phases.

Both TG and DSC studies require sample mass as input. The appropriate weighing was performed using a Mettler-Toledo AT261 Delta Range analytical microbalance (Mettler-Toledo GmbH, Greifensee, Switzerland) with a resolution of 0.01 mg. The same balance equipped with a density measurement kit was used to determine the density, or more precisely, the effective (overall) density of the tested composite structures. These measurements were performed using the buoyancy technique, with distilled water applied as an immersing fluid. The density *ρ*_0_ = *ρ*_0_(*T*_0_), where *T*_0_ stands for the room temperature (RT), was determined for specimens prepared for the thermal diffusivity test. These samples are large enough to obtain relatively representative density values despite macroscopic structural inhomogeneities.

TG 209 F3 Tarsus thermobalance, NETZSCH-Gerätebau GmbH, Selb, Germany, was used to conduct thermogravimetric tests (TG). The measuring range of the thermobalance covers the temperature range from room temperature (RT) to 1000 °C, the resolution is 0.1 μg, the maximum weight range is 2000 mg, the rates of temperature change from 0.001 °C/min to 100 °C/min, and the capacity of a standard alumina sample pan is 85 μL. Due to the lack of precise information on the composition of the material and the inhomogeneity of the structure, it was decided to set the upper temperature of the furnace heater (“STC off” mode) at 600 °C and the rate of temperature change at 10 K/min. Such settings mean a slightly lower value for the maximum temperature and slightly lower values for the rate of temperature change during heating. A control system was used to manage the furnace heater, and the flow rates for both the nitrogen gas in the chamber and the protective zone gas were set at 20 mL/min.

**Figure 1:**
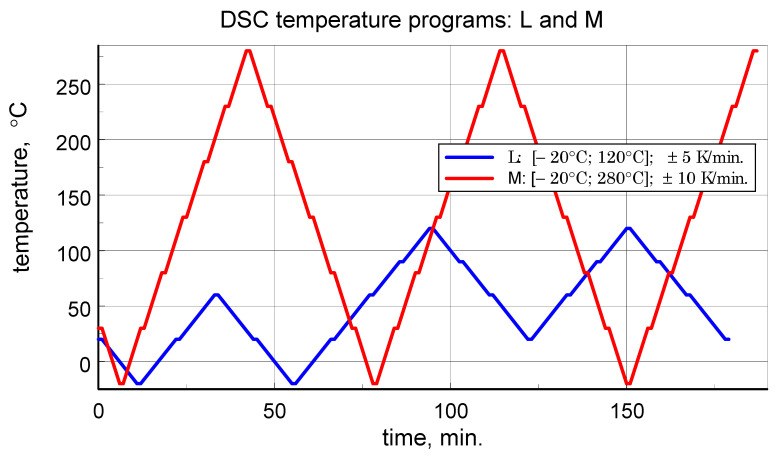
Temperature scans programs of low temperature range test L and medium temperature range test M, with repeated heating and cooling cycles.

Dynamic Scanning Calorimetry (DSC) microcalorimetric studies were performed using a Pyris 1 power-compensated scanning microcalorimeter from PerkinElmer, Inc., Waltham, MA, USA with a temperature range of −30 °C to 600 °C or from room temperature (RT) to 710 °C and a claimed accuracy of enthalpy and specific heat determination of ±2%. Attention in the study was focused on the determination of specific heat. The three-curve method ([12,14,15,16]) was used to determine the specific heat. The study employed a dedicated, author-developed temperature variation program, previously described in the literature [6,13], which enables obtaining reliable results in both heating and cooling modes of microcalorimeter operation. Each sample was tested first according to the thermal program with the maximum exposure temperature limited to 120 °C (denoted as program L), and then according to the program specified as medium-temperature M (extended range). The low temperature program corresponds to the typical operating temperature range of aircraft composites. The basic data for the programs are provided in Table 1, and the temperature changes over time for both L and M programs are illustrated in Figure 1.

Complementing the study of thermophysical properties were measurements of thermal diffusivity, i.e., the ratio of thermal conductivity to volumetric heat capacity. A modified temperature oscillation method was used to determine this parameter. The author’s modifications of the Ångström method [17] involve considering a finite characteristic dimension of the investigated object–a plate [18] and introducing a linear temperature increase as the basis for the oscillations [19]. The temperature oscillation can be applied, preserving high temperature resolution of measurements [6] performed at scanning mode operation and distinguishes the measurement method from other methods, such as laser flash analysis (LFA), giving point-like results in the temperature domain [20,21]. A description of the test stand, together with a presentation of the procedures for testing directional properties, is discussed in publications [19,22,23]. Processing of the measurement signals results in two independently calculated values of thermal diffusivity: one determined by comparing the amplitude of the periodic temperature change excitation signal and the periodic response, the so-called amplitude value *a*_ψ_, and the other, determined by the phase shift, the phase value *a*_φ_. In the model case of one-dimensional heat flow, without convective losses from lateral surfaces, both values should be equal. That allows for a preliminary check of the correctness of the measurement.

**Figure 2:**
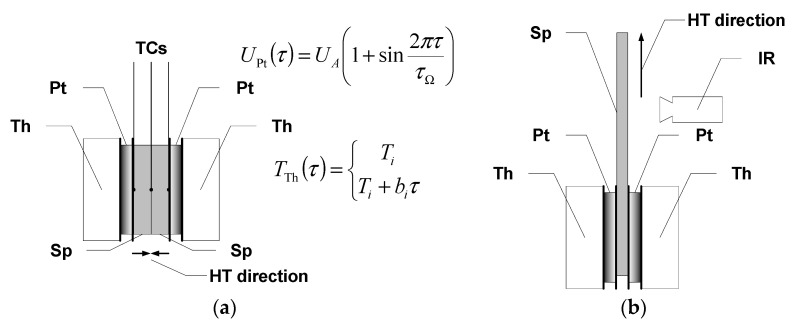
Arrangement of the measuring head for: (**a**) out-of-plane thermal diffusivity and (**b**) in-plane thermal diffusivity investigation of anisotropic material specimens (Sp–specimen, Pt–Peltier element, Th–thermostatic block, TCs–thermocouples, IR–infrared camera). In the figure, formulae for the voltage supply of the Peltier semiconductor element and temperature regulation of the thermostatic blocks are also provided.

The thermal diffusivity of the composite structures was determined by considering their directional property differences. For measurements in the direction perpendicular to the surface (transversal/out-of-plane component), square-shaped specimens with a side length of 40 mm were used, while specimens with a length of about 60 mm and a width of about 10 mm were used to measure the thermal diffusivity in the longitudinal direction (longitudinal/in-plane component). In the case of measuring transverse diffusivity, an outside bilateral symmetric temperature oscillation excitation was used with the measurement of the response signal at the contact surface of two samples put together with square surfaces, i.e., the symmetry surface of the system. In this case, the characteristic dimension for calculating the thermal diffusivity was equal to the sample thickness, i.e., approximately 6.2 and 5.3 mm for epoxy-mat composites and approximately 3.5 mm for epoxy-fabric composites. In Figure 2, the arrangement of the measuring head for out-of-plane and in-plane thermal diffusivity is shown. The measurement system makes it possible to perform thermal diffusivity measurements in the range from about −10 °C to about 100 °C. The period of excitation was selected each time according to the properties of the tested material and ranged from 30 s for graphite composites to 60 s or 120 s for glass composites.

Measurements of the longitudinal component of thermal diffusivity were performed with unilateral excitation and using an infrared camera to record temperature changes (Figure 2b). Due to the time-consuming nature of image data processing, the measurement range was limited to room temperature, considering that the temperature dependence is well characterized by the data from the transverse component of thermal diffusivity tests. It should be mentioned that the specimens were tested without removing the paint coating, and the registration of temperature changes was made from the opposite side. To carry out the measurements, the surfaces of the samples were coated with a layer of GRAPHIT 33 flake graphite with a thickness of no more than 20 μm. Changes in the temperature distribution were recorded using a Flir SC5600 thermal imaging camera (FLIR Systems, Inc., Wilsonville, OR, USA) with a recording frequency of 1 Hz for a 120 s excitation period and 0.25 Hz for a 240 s excitation period. For data processing, temperature change signals were collected as the spatial mean temperature of the control lines. The control lines were declared along with the expected isotherms. The lines were spaced at approximately 2.5 mm intervals.

**Figure 3:**
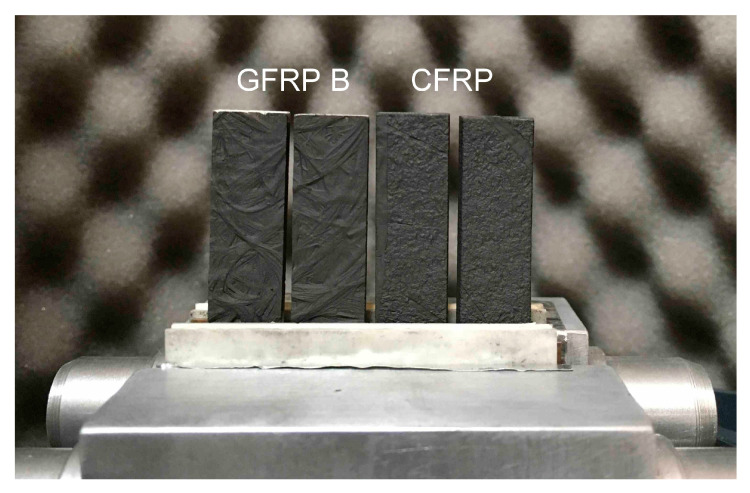
View of samples of composite structures mounted in a holder for recording temperature changes with a thermal imaging camera in measurements of the longitudinal component of thermal diffusivity: from the left, two specimens of GFRP B, on the right, two samples of CFRP.

**Figure 4:**
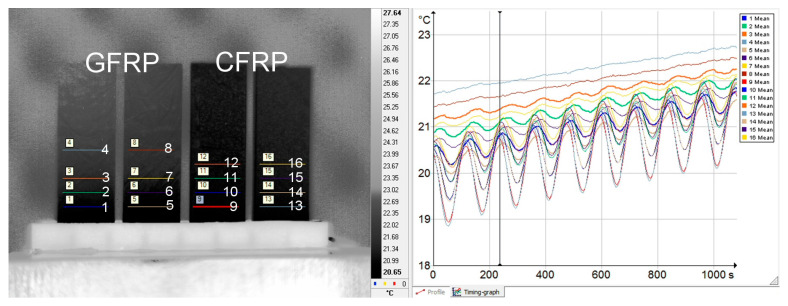
Image (screenshot) from the thermal imaging camera with plotted readout lines (indicated by subsequent numbers) of temperature change signals (**left**) and illustration of recorded temperature changes (**right**).

The study of the longitudinal component of thermal diffusivity was carried out in two stages: separately for the GFRP A sample, and separately for the GFRP B and CFRP samples. The reason was that the thickness of the GFRP A sample differed too much from the others. An example of the arrangement of samples in the measuring head is shown in Figure 3. In turn, the image of the temperature distribution recorded during the measurements and the result of reading the temperature changes are illustrated in Figure 4. The temperature change signals shown in this figure include, among other things, the values of the average line temperature read from the control lines visible on the thermogram. In determining the position of each line, the uniformity of the spatial distribution was checked, which is an indication of one-dimensional heat flow, a condition for methodological correctness.

The distances in mm between the lines were determined photogrammetrically. Thermal diffusivity values were calculated for pairs of signals, one of which was the excitation, and the other was the thermal response. The distance from the edge of the end (top) of the sample was also included in the calculations.

The thermal diffusivity measurement data were processed according to the procedures described in [19,23]. In the case of convective heat losses, the phase thermal diffusivity *a*_φ_ value is the upper limit, and the amplitude *a*_ψ_ value is the lower limit of the sought thermal diffusivity value. The sought value can be determined as the geometric mean of the two above-mentioned [17,19].

## 3. Results

### 3.1. Macrostructure, Microstructure, and Effective Density

**Figure 5:**
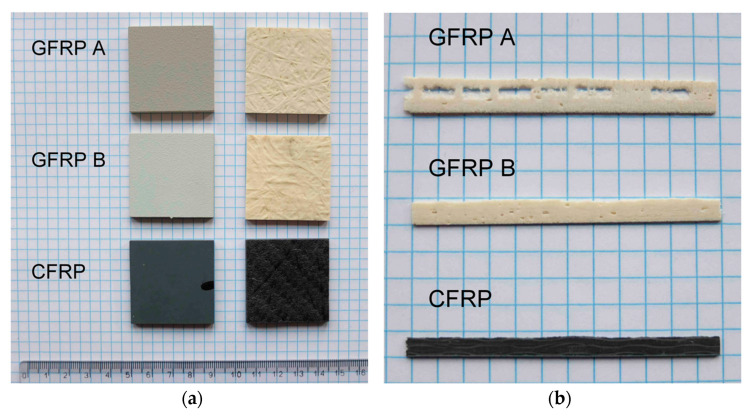
View of samples taken from individual plates of composite structures ((**a**); ruler scale in cm) and side view of samples (**b**).

Illustrative photographs of the tested composites are depicted in Figure 5. Figure 5a shows the surfaces of the upper and lower surfaces. All tested specimens were coated with factory paint on one side (grey surfaces visible in the left column of Figure 5a). Figure 5b represents a macroscopic cross-sectional view of the original specimen from which the samples were cut.

**Figure 6:**
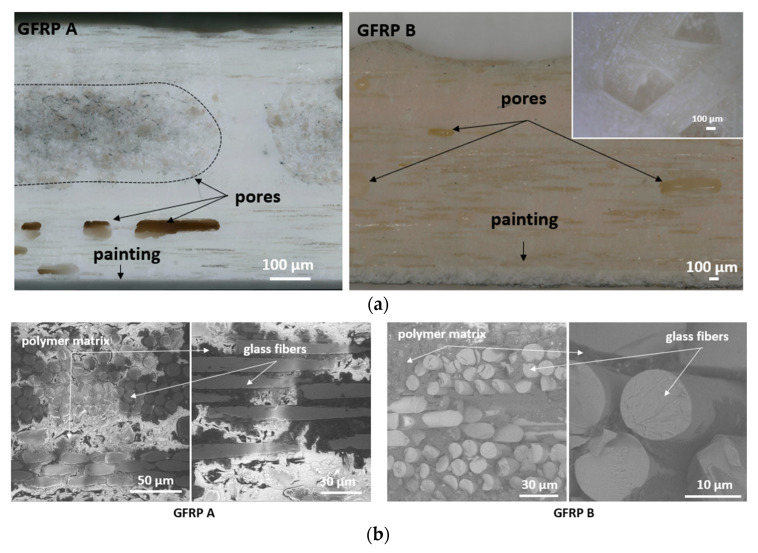
Cross-sectional view of GFRP A and GFRP B macrostructure, together with the view of the surface of the epoxy-glass composite GFRP B (**a**) as well as SEM image of the microstructure of GFRP A and GFRP B (**b**).

Figure 6 shows cross-sectional views of GFRP A and GFRP B, highlighting their macrostructures with pores of varying shapes and sizes, concentrated mainly in interlayer spaces. Larger pores form clusters, while smaller spherical voids are distributed more evenly. Two porosity types were identified: cylindrical voids between fibers and spherical voids between fiber bundles. In GFRP A, large subsurface pores were observed, often lacking glass reinforcement, whereas GFRP B revealed non-uniform fiber distribution and visible epoxy particles. Higher-magnification images of GFRP A and B microstructures are provided in Figure 6b.

**Figure 7:**
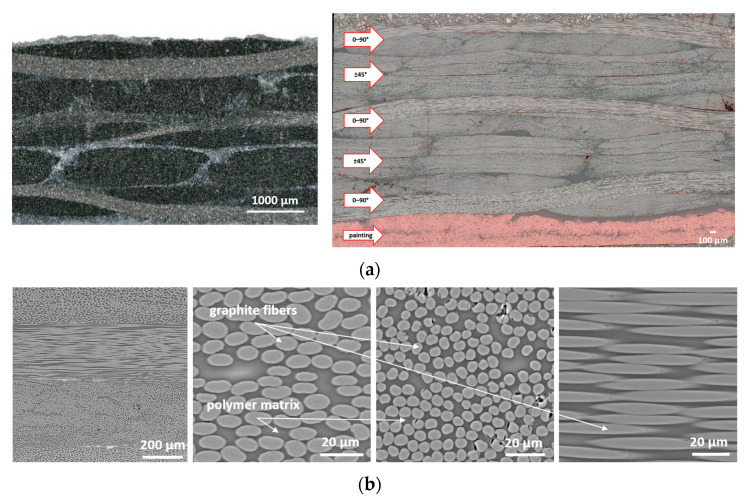
Macrostructure of CFRP (**a**) lower magnification (**left**) and image taken on a specimen cut at an angle of 45° (**right**) (**a**), as well as SEM images of the microstructure of the CFRP (**b**).

Figure 7a presents CFRP microstructures, confirming the layered interlacing of woven carbon fabric. Fibers oriented at different angles, resin-rich zones, and variations in fiber density are visible. Figure 7b shows SEM images, revealing uniform fiber distribution within bundles, low porosity (Vv < 0.2%), and a high fiber volume fraction (Vf ≈ 57%), with graphite fiber diameters of 5–6 µm. Minor defects such as pores, broken fibers, and detachments were also observed.

**Figure 8:**
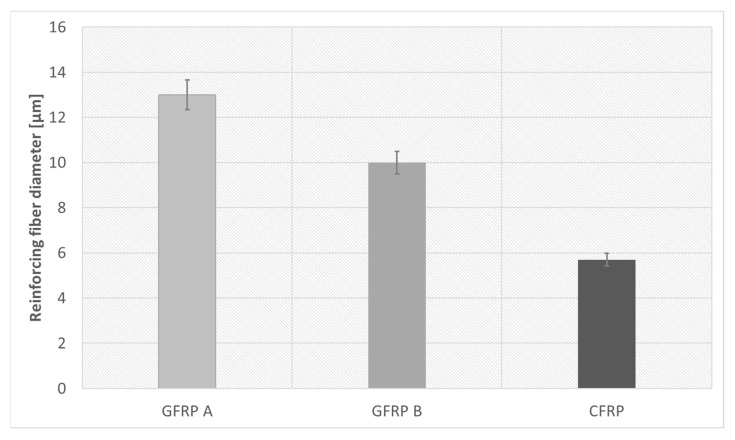
A graph showing the diameter of reinforcing fibers for each composite.

Figure 8 compares fiber diameters, showing GFRP A with the largest and CFRP with the smallest. Effective density studies, performed using the buoyancy method on samples prepared for longitudinal diffusivity tests, are summarized together with porosity data in Table 2.

Additionally, taking into account density and determined volume fraction of the components of tested composites (Table 3), the mass fraction of reinforcement was calculated by using

(1) $$\omega_{r}=\frac{V_{r}\rho_{r}}{V_{r}\rho_{r}+\left(1-V_{r}\right)\rho_{m}},$$

where $\omega_{r}$ is the mass fraction of reinforcement, *V*_r_ is the volume fraction of reinforcement, $\rho_{r}$ is a reinforcement density, $\rho_{m}$ is a matrix density. The density data for the components were assumed: for resin based on [24], glass fibers on [11], and graphite fibers on [25].

### 3.2. Thermophysical Properties

The analyses carried out in Section 3.1 identified structural anisotropy and the associated expected variation in directional properties in two main characteristic directions: in the direction perpendicular to the plate surface, i.e., traversal or out-of-plane, in other words, and in the longitudinal, i.e., in-plane direction. In the case of glass composites, due to the stochastic variation in the longitudinal directions of the arrangement of individual mat fibers, all longitudinal characteristic directions appear to be equal. When studying graphite composite, the direction of the cut of the specimens for longitudinal characteristic testing may be of importance. This is determined by the regular orthogonal structure of the carbon fibers. The thermal behavior of composites is highly dependent on fiber arrangement, a fact underscored by [26]. Composites with aligned fibers show a strong directional dependence of thermal properties, while chopped fiber composites, though more uniform, exhibit localized fluctuations in thermal diffusivity.

While the anisotropy of the tested materials, as a typical feature of most composite structures, should not pose major research problems, two geometric-structural features that prevent the direct application of standard materials testing procedures were recognized at the outset of the visual evaluation. The first of these features is the variation in the structure and surface condition of the face of the plates concerning the inner surface (Figure 5a). This is related not only to the presence of the paint layer but also to the variation in topography: the inner surface of each structure shows greater irregularity. In this case, it is necessary to take this fact into account when interpreting the test results, and what is particularly important, to distinguish the result of the measurements from the actual material property [22]. The second problem relates to local differences in the thickness of the plates supplied for testing. In the case of the GFRP (Glass Fibred Reinforced Polymer) composite structure, this is compounded by high porosity with an irregular distribution of gaseous inclusions near the inner surface (Figure 5b). The variation in slab thickness and the presence of unevenly distributed pores are accompanied by local variations in composition. In the homogenization interpretation, the studied structure is composed of locally differentiated materials. Given this, the determination of the actual properties becomes problematic. Only the possibility of determining representative properties remains, and only for selected test cases. However, the results obtained should be fully useful both for characterizing the structure and for carrying out structural calculations, also taking into account operational recommendations. The reliability of the data in this regard depends on the accurate demonstration of metrological limitations. The two primary objectives of the study were:

1. Providing material data in terms of the thermodynamic (thermophysical) parameters studied for engineering calculations;
2. Determination of thermal stability and determination of parameters of characteristic transformation of the matrix material of the studied composite structures.

The thermal behavior of composite materials is a critical factor in their performance, particularly in demanding applications like aerospace. Consequently, thorough thermophysical testing is indispensable. While methodologies employing techniques such as DSC, Laser Flash Analysis (LFA), and Dynamic Mechanical Thermal Analysis (DMTA) have been proposed [27], comprehensive studies in this area remain relatively limited.

### 3.3. TG/DTG Thermogravimetric Studies

**Figure 9:**
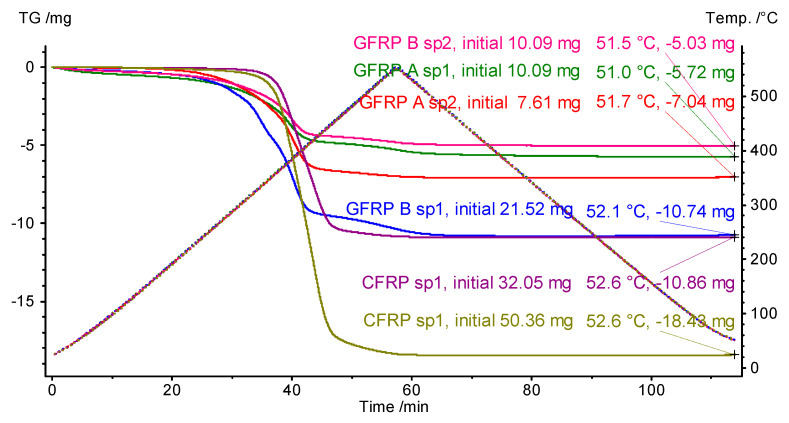
Temperature changes of the tested samples during the TG test (dashed lines, right axis of ordinate) and a summary representation of the test thermograms of all samples with a list of global mass changes (Table 3) (‘sp1’ and ‘sp2’ denote specimen 1 and specimen 2, respectively). Total mass loss data are presented in the order of appearance of the TG curves at the end of the experiment.

The weight data of the samples are included in Table 4. The table also includes the results of measuring the total change, in the case of a loss of the weight of the samples tested. A single measurement consisted of subjecting the test sample to heating and then cooling according to the temperature variation program shown in Figure 9.

Assuming that the epoxy resin material is completely decomposed into released gaseous products, the result of the final mass measurement allows the determination of approximate values of the mass shares of the filling material. The approximate value of the mass share of graphite in the CFRP structure is about 65 wt.%, and of glass mat fibers in the GFRP B structure is about 50 wt.%. Determining the mass share of glass in the GFRPA composite to be about 40 wt.% may be considered problematic, but the sheer fact of the lower fill content in this structure is highly probable.

**Figure 10:**
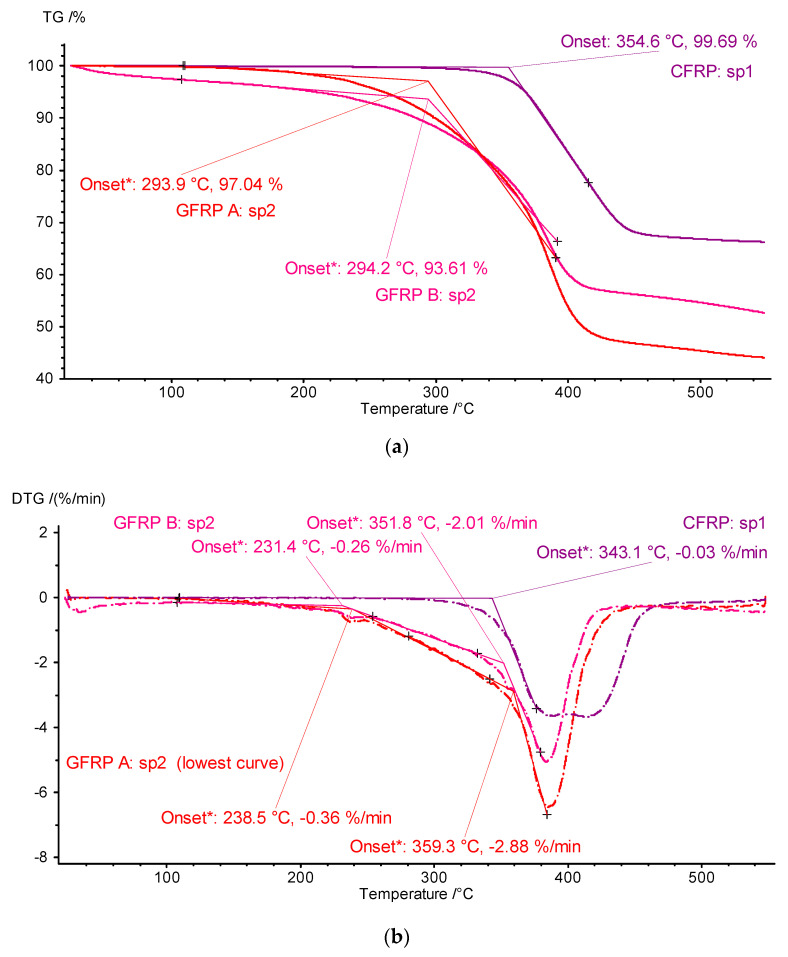
Thermograms of TG measurements and the results of their post-processing in the form of determined DTG signals and ONSET temperature values: for TG (**a**) and DTG (**b**) curves (‘sp1’ and ‘sp2’ denote specimen 1 and specimen 2, respectively).

To develop the results, TG and differential thermogravimetry (DTG; differential: derivatives of mass changes after time) thermograms of the key first heating stage plotted as a function of temperature were prepared (Figure 10). The results of developing the TG and DTG signals are shown in Figure 10a and Figure 10b, respectively. The TG waveforms visualized in Figure 10 show a qualitative similarity in the mass changes-matrix phase distribution-for the tested samples of the structure GFRP A and GFRP B, along with similar values of the temperature of the conventional onset of the ONSET transformation for the TG curves of approximately 294 °C, and small but clear differences in the mass changes of the graphite CFRP composite compared to the glass composites and a significantly different ONSET TG temperature of approximately 355 °C.

The results of in-depth analyses carried out for DTG signals bring to light the fact of the two-stage transformation of matrix material decomposition. In the case of glass composites, the process starts at a lower temperature value (ONSET DTG about 295 °C) and initially proceeds smoothly, and then, above about 350 °C, it accelerates until complete decomposition in the vicinity of 440 °C. (Figure 10, GFRP A sp2 and GFRP B sp2 curves). The two-stage transformation is a characteristic of the graphite composite structure matrix, i.e., epoxy resin decomposition. The temperature at the beginning of the transformation is about 340 °C, and the end is about 460 °C.

Given the differences in the ONSET TG and ONSET DTG temperature values, the lower values of the DTG parameters: 230 °C for the GFRP structure and 340 °C for the CFRP structure, should be taken as the basis for determining the limits for other measurements and setting possible operating limits.

### 3.4. Microcalorimetric Studies

In light of the results of the structure evaluation, because of the high porosity and heterogeneity of the GFRP A structure, microcalorimetric measurements were limited to the study of GFRP B and CFRP structures. Due to the small size of DSC samples, obtaining reliable qualitative results for heterogeneous materials requires laborious repetitive testing of many separate samples. The parameters of the GFRP B structure, as a representative glass, should not generally differ from the properties of the GFRP A structure. This is indicated by the convergent results of TG measurements of both structures.

**Figure 11:**
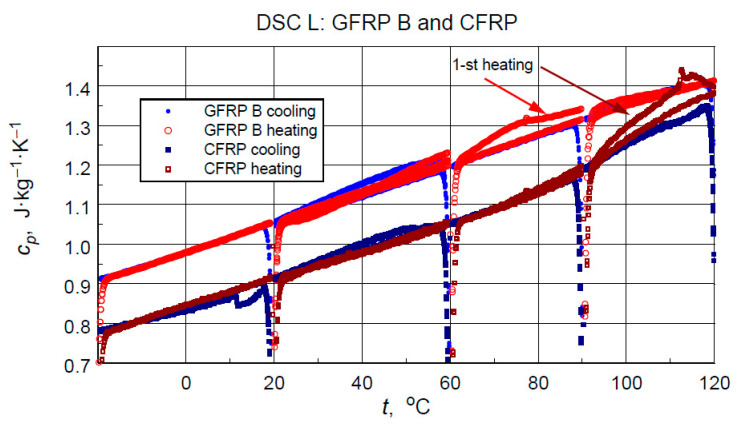
Direct results of the DSC Heat Flow thermogram processing of the low-temperature test into specific heat (with artefacts of irregular heat transfer effects at the beginning of each heating/cooling ramp).

**Figure 12:**
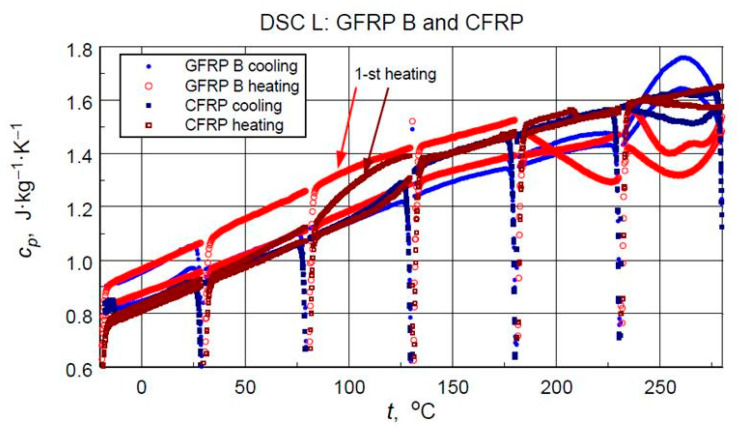
Direct results of the DSC Heat Flow thermogram processing of the medium temperature range test into specific heat (with artefacts of irregular heat transfer effects at the beginning of each heating/cooling ramp).

The direct results of developing the thermograms into calculated specific heat values using the three-curve method for the low-temperature measurements are illustrated in Figure 11. Figure 12 compares the results of the high-temperature tests. Analyzing the results of the low-temperature measurement shown in Figure 11, the following can be concluded:

i. As expected, the higher specific heat values of the glass-filled structure sample compared to the graphite structure. This is determined by the higher specific heat of glass than the specific heat of carbon [11] and the higher values of the mass share of graphite filling shown by thermogravimetric studies.
ii. Stability and compatibility of thermal properties of the tested structures in the range of low-temperature excitation.
iii. Possible minor effects of moisture or another volatile agent, for example, excess hardener release marked in the form of slightly higher specific heat values in the first heating cycles in the temperature range above 60 °C for glass composite and 90 °C for graphite composite. In addition to the above-mentioned effects, these phenomena may be caused by hardening effects or thermally induced irreversible structural changes.

From the point of view of operational requirements, the most important thing is the repeatability of the results of testing the properties of a material subjected to cyclic thermal loading. The results of the medium-temperature tests (M) are unfortunately burdened by mass loss effects. Nevertheless, they provide an opportunity to determine the correspondence of the determined values of specific heat in the initial phase of the medium-temperature cycle with the results of an earlier low-temperature measurement.

**Figure 13:**
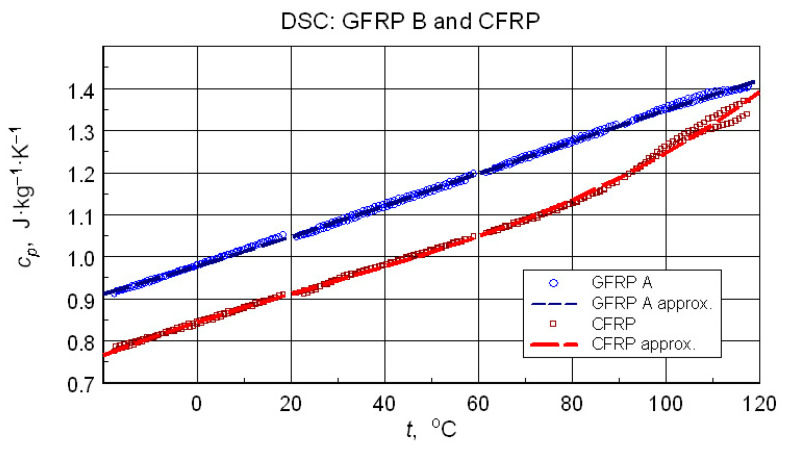
Results of a B-spline approximation of the calculated specific heat capacity values recommended for characterization of the investigated composite structures.

Direct results, especially in the form of raw thermograms, are a good illustration of metrological problems of testing real composite structures. However, even in such a situation, it is possible to estimate some representative and reliable data. In the case of the TG test, this was information on the composition of the samples tested. If DSC data are taken into account, it is even recommended to present representative *c_p_* data for calculating the tested samples’ thermal conductivity based on the results of the thermal diffusivity measurement. In the discussed case, such data were obtained by a third-degree polynomial approximation

(2) $$c_{p}\left(t\right)=\sum_{i=0}^{n}\mu_{i}t^{i}$$

of the specific heat values obtained for 2nd cycles, i.e., the state of thermally stabilized samples in the range of thermal load. The results are shown in Figure 13. The numerical data of representative temperature dependencies are presented in Table 5.

DSC test results often form the basis for determining the glass transition temperature of the matrix material–epoxy resin. This applies to both the isolated component [24] and the composite structure sample [23]. In this case, the glass transition can only be clearly identified in the CFRP structure. Analysis of the data in Figure 13 allows us to provide an approximate Tg value of approximately 80 °C. The test results for the GFRP B sample do not allow for the identification of a glass transition, although its occurrence is virtually certain. More sensitive test methods, such as dilatometry or DMA, would be necessary to determine this transition.

### 3.5. Thermal Diffusivity

**Figure 14:**
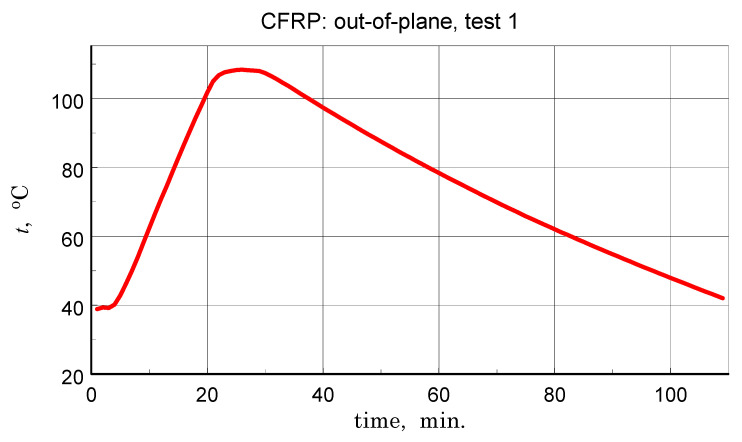
Temperature program of the first measurement of the GFRP structure tests-component of the linear scanning of the temperature interval on which oscillations with an amplitude of about 1 K are superimposed (see [19]).

**Figure 15:**
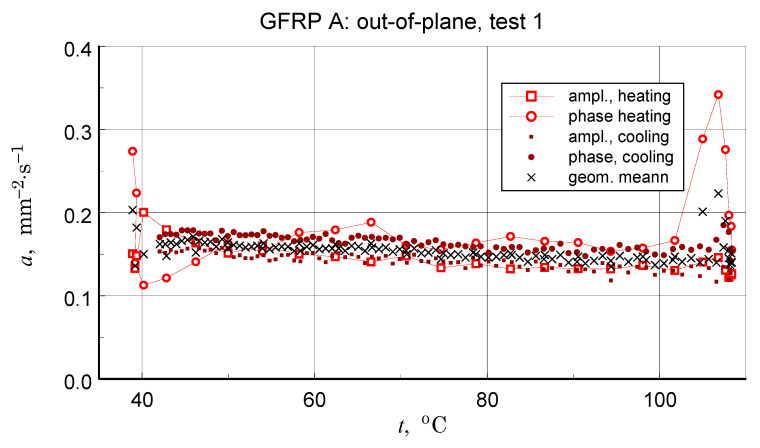
Results of the first measurement performed for the GFRP A structure-illustration of the magnitude of differences between the amplitude and phase results, the effects of disordered heat transfer (peaks for the extremes of the temperature interval), and the geometric mean data used to determine representative characteristics.

**Figure 16:**
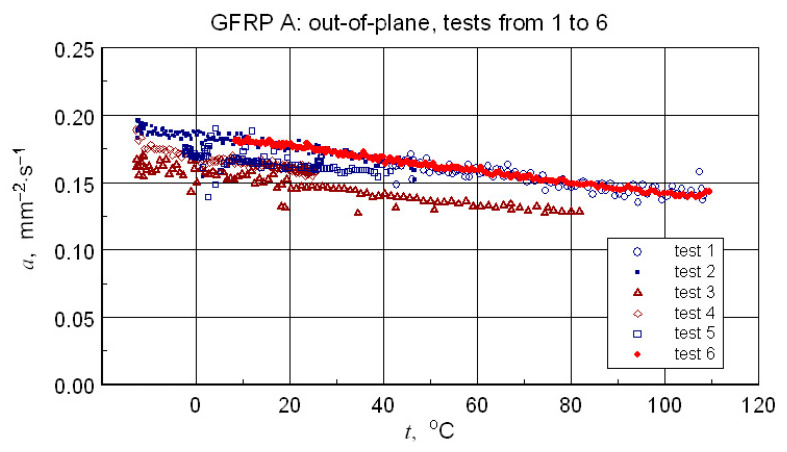
Results of all six GFRP A structure tests-illustration of the differences in results due to changing the configuration of the samples.

**Figure 17:**
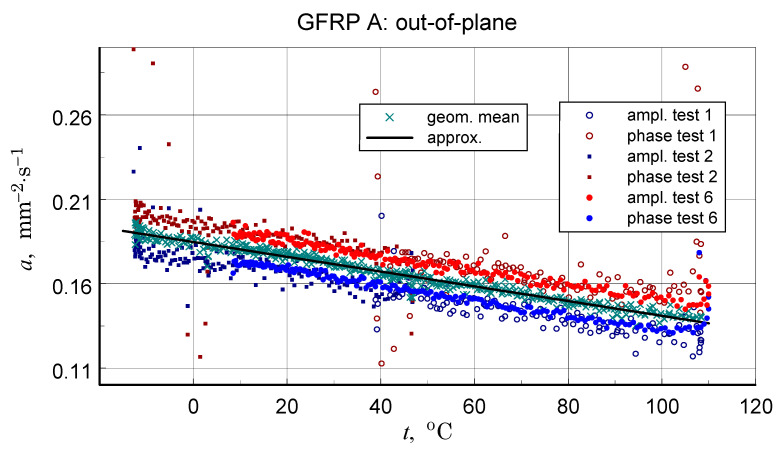
Results of measurements used to determine a representative transversal thermal diffusivity-temperature relationship for the GFRP A structure, input data for calculations, including the geometric mean, and resulting approximation of these data.

Before discussing the obtained thermal diffusivity measurement results, it should be noted that due to the non-homogeneous structure of the tested materials, the presented results should be treated in terms of effective diffusivity. The results of the transverse diffusivity measurement of the GFRP A structure are shown in Figure 14, Figure 15, Figure 16 and Figure 17. In light of the phenomena identified and characterized in the previous sections of the differences between the first and subsequent thermal loading cycles, it is important to pay attention to the results obtained in the first measurements. The temperature program of the first measurement of the structure in question is shown in Figure 14. The results of this measurement, illustrated in Figure 15, do not show clear signs of phase transitions, residual polymerization effects, or moisture release: the heating and cooling characteristics of the sample (in fact, the two samples) coincide. This provides a basis for using the geometric mean of the amplitude and phase thermal diffusivity data to determine a representative relationship (Refs. [17,19]).

Repeating the measurements for a changed orientation of the sample set highlights possible differences in the measurement results (see Figure 16). Treating the obtained results as a measure of uncertainty, a decision was made to select results that were quantitatively consistent for determining a representative approximation characteristic for the longitudinal diffusivity of the GFRP A composite.

In light of the presented considerations, the results of the geometric mean-square approximation of the geometric mean data of the calculated amplitude and phase values can be considered as an upper limit for the analysis. The baseline (ampl. and phase), input data, and calculation results are illustrated in Figure 17.

**Figure 18:**
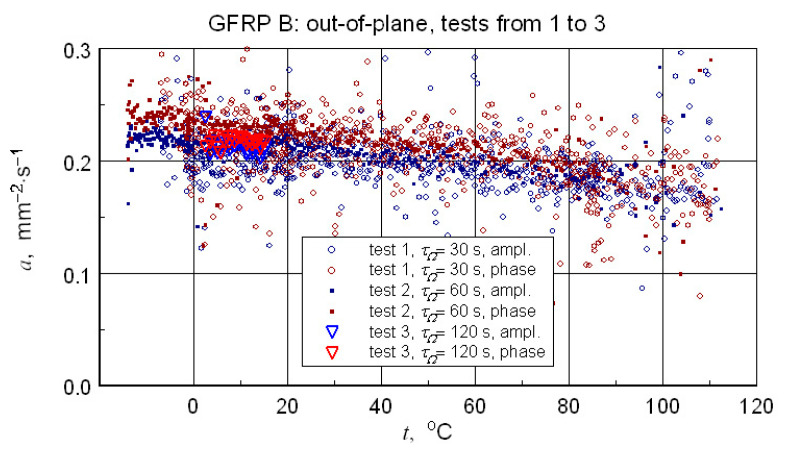
Summary of the results of thermal diffusivity tests of the transverse component-for samples of the GFRP B structure.

**Figure 19:**
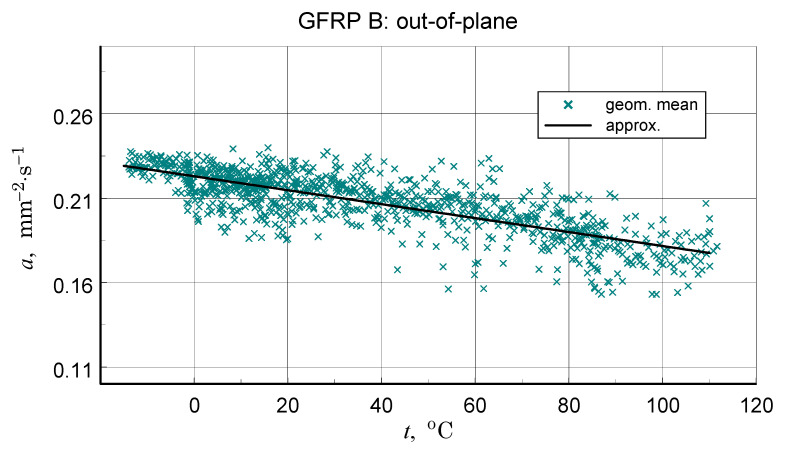
Data for the determination and resulting approximation characteristics of the thermal diffusivity of the transverse structure of GFRP B.

**Figure 20:**
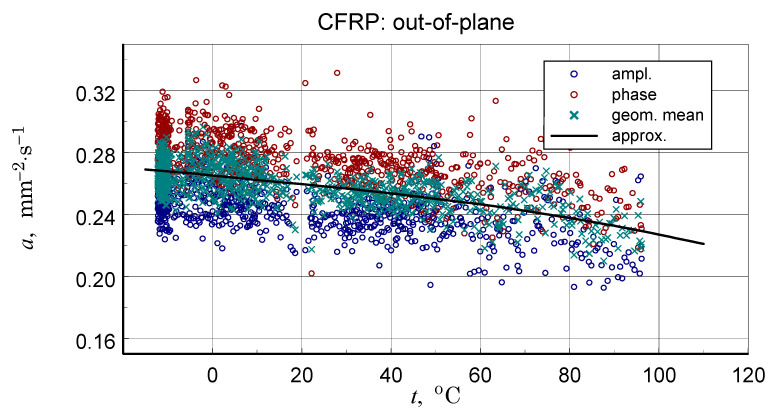
Summary of measurement results (ampl., phase), data for determination (approx.), and resulting approximation characteristics of thermal diffusivity of transverse CFRP structure.

**Figure 21:**
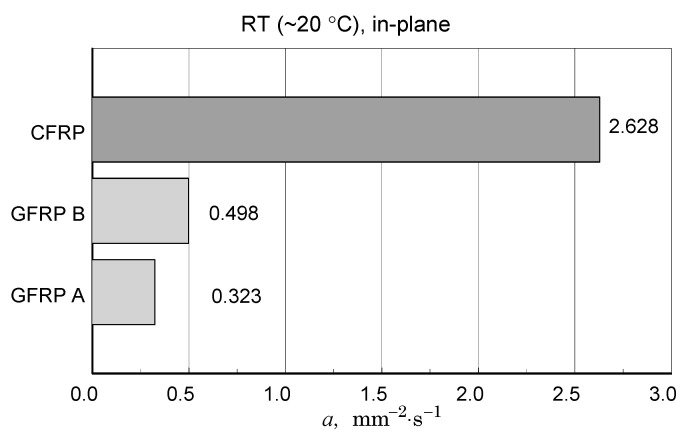
Comparison of longitudinal thermal diffusivity values of the tested composite structures.

A summary of the transverse component of thermal diffusivity measurement results of the GFRP B structure is illustrated in Figure 18 and Figure 19, while the thermal diffusivity results of the CFRP structure tests are shown in Figure 20. Furthermore, the determined average values of the longitudinal component of thermal diffusivity are shown in Figure 21. They correspond to room temperature, but the temperature dependence of this component can be deduced from the transverse thermal diffusivity component.

The representative longitudinal thermal diffusivity characteristics shown in Figure 17, Figure 19 and Figure 21 were determined by fitting the given geometric mean values

(3) $$a=\sqrt{a_{\psi }a_{\phi }}.$$

The same as for the DSC data polynomial approximation function was used (Equation (3)). For GFRP composites, data linear approximation was utilised for the CFRP data cubic. The coefficients of the approximation polynomials are given in Table 6. Table 7 shows the results of the longitudinal diffusivity tests.

The CFRP composite showed the highest transverse and longitudinal thermal diffusivity. For the GFRP A and GFRP B, longitudinal diffusivity was roughly double the transverse diffusivity, but for the CFRP, this ratio was ten times higher.

### 3.6. Thermal Conductivity

The experimental results presented above primarily illustrate the possible variability of measurement results for the properties of real, i.e., utility material composite structures. This was demonstrated, among other things, by presenting multiple thermal diffusivity studies. However, the obtained data can be used to estimate thermal conductivity values. Given the variability of results, structural irregularities, and the influence of thermal history, as demonstrated by TG and DSC studies, these values will be indicative. Moreover, they will be effective, resulting from the parameters of individual structural elements. However, presenting the relevant numerical data will provide data for potential comparative analysis. However, to determine data for the GFRP A structure, it is necessary to make assumptions regarding the specific heat capacity. Due to the significant structural heterogeneity, determining this quantity through experimental testing would require measurements for many material fragments to obtain statistically representative results. Such testing was not planned within the ongoing program. Therefore, it was assumed that the GFRP B structure data would be used to determine approximate values of the resultant thermal conductivity of the GFRP A structure. The results of calculations of the effective thermal conductivity of the tested structures, obtained as

(4) $$k_{\mathrm{eff},\mathrm{RT}}=\rho \cdot a_{\mathrm{eff},\mathrm{RT}}\cdot c_{p,\mathrm{eff},\mathrm{RT}},$$

where *k* stands for thermal conductivity and subscripts eff and RT indicate the effective character of the parameter and room temperature, respectively, are presented in Table 8. The calculations were based on data representative of the room temperature characteristics indicated in Table 6 and Table 7, as well as on the density data from Table 2. By using the term representative, we emphasize the fact that these values are valid for the thermally stabilized state, as in the case of DSC test results.

## 4. Discussion

The results of macrostructural studies already indicate significant heterogeneity in the tested composite structures. This is clearly a result of imperfections in the manufacturing process. Therefore, any data from quantitative analysis is more illustrative. This is particularly true for studies using small samples: TG and DSC. Nevertheless, with a relatively wide tolerance range, the obtained data allow for the characterization of the tested structures. The scatter in the results illustrates possible deviations between the actual structural data and the model materials produced solely for property testing. In this context, the presented results are important from a metrological perspective.

Despite this problem, it is possible to conduct a comparative pooled analysis. The already published results from [24] for GFRP and from [21,22] for CFRP have been taken as a reference for this analysis. At this point, however, it is worth noting a significant difference between the GFRP structure we investigated and the structure described in [23]: in our case, the longitudinal glass fiber additions were of a disordered nature, while the literature data refer to a structure with a glass fabric filler with a regular weave of fibers arranged at right angles. The already published results from [23] for GFRP and from [21,22] for CFRP have been taken as a reference for this analysis (Table 9).

As shown in Table 2, the density measurement results for the tested structures are consistent with the structural analysis results: the presence of a large number of macroscopic pores in GFRP A significantly reduces the effective density. The density measurement results for GFRP B and CFRP structures are generally consistent with the literature data (Table 9) and reflect the effect of the higher density of glass compared to the density of graphite.

Comparison of the investigated structures’ composition estimation results based on structural studies (Table 3) with the TG test data (Table 4) reveals significant discrepancies for GFRP structures. The residual mass data from Table 4 can be treated as approximate mass composition data. As shown by the TG test results from [21], the effect of mass reduction of resin-based structures at temperatures above 600 °C is negligible. In our study, repeating the measurements did not reveal significant changes in the mass of the tested formulation. However, independent data on the mass fraction of components can be obtained by analyzing the specific heat (in practice, effective heat capacity *c_p_*_,eff_) using the homogenization rule

(5) $$c_{p,\mathrm{eff}}\left(t\right)=\sum_{i=0}^{n}g_{i}c_{p,i}\left(t\right)$$

where *g_i_* stands for the mass fraction of the component *i*. Assuming that the mass fraction of the gas is negligible and that the specific heat of the components is known, the Equation (4) relationship together with the closing equation

(6) $$1=\sum_{i=0}^{n}g_{i}$$

allows the determination of the mass fractions of the components. Results of the appropriate calculations performed with the epoxy resin data after [24] and the glass and carbon data after [11,25], respectively, are shown in Table 10. Due to the low representativeness of GFRP A samples caused by structural inhomogeneities, this composite was excluded from both the DSC tests and this comparison. As can be seen, the greater imperfections in the GFRP microstructure compared to CFRP result in greater scatter in the analyzed data. However, considering the lack of systematic study results, the agreement between the composition data can be considered satisfactory. The agreement with the CFRP test data is good.

Comparing the results of structural, TG, and DSC studies allows for another methodological observation. While in thermal analysis studies, the small sample sizes are partially compensated by the volumetric nature of the studies, in the case of cross-sectional studies, the limitation results from the unrepresentativeness of the selected image for the entire volume.

When analyzing the test data on specific heat and directional components of thermal diffusivity and their temperature dependence, attention should be paid to the influence of structural inhomogeneities and thermal state history. As shown in Figure 12, differences in specific heat values caused by composite annealing can reach 15% of the initial value. The effect of temperature exposure or ageing on changes in composite properties is known [1,5], but the data presented here illustrate the possible scale of changes.

The numerical effective specific heat measurement data (Table 5, Figure 13) can be considered qualitatively consistent with the literature data (Table 10). The quantitative differences are not surprising in light of possible differences in the properties of the components of different composite structures.

In light of the structural imperfections demonstrated in the structural studies, the qualitative consistency, but also the satisfactory quantitative consistency of the thermal diffusivity measurement data (Table 6 and Table 7) with the literature data (Table 9) may be surprising. It appears that the obtained results not only accurately reflect the directional differences in properties but also maintain the correct value proportions. It should be emphasized, however, that structural inhomogeneities result in differences of up to 20% in the values of the tested parameter (see Figure 16). In each case, however, the temperature dependence of thermal diffusivity is accurately determined. This is a result of the high temperature resolution of the studies.

In concluding the comparative analysis of the obtained results, attention should be paid to the consistency of the TG results, in particular DTG, DSC, and high-resolution temperature diffusivity measurements, in identifying differences in the matrix properties of GFRP and CFRP composites.TG studies revealed both differences in the onset temperature of phase transformations (Figure 10a) and the decomposition process (Figure 10b). Differences also occur in the low-temperature region. In this range, the temperature dependence of the effective specific heat and thermal diffusivity demonstrates consistent evidence of glass transition effects in CFRP composites (Figure 13 and Figure 20), identifiable by inflections in the curves after exceeding 80 °C. The temperature dependencies of the thermophysical parameters of GFRP (Figure 13 and Figure 19) do not show such phenomena in the area up to 110 °C. This does not mean that such a transformation does not occur, but its identification requires further research. Similarly, it would be advisable to determine the individual properties of the components of the three structures: the matrix and the filler materials. In the presented situation, testing a composite taken from a finished product is not possible. For this reason, the values presented in Table 3 and Table 10 were estimated based on approximate data, identical to those for the matrix material. This fact, however, does not undermine the qualitative and methodological conclusions. In particular, the benefits of comprehensive studies with high temperature resolution [6] and the use of the same methods for studying directional properties are fully justified. Due to differences in characteristic dimensions, this is not always possible [15].

## 5. Conclusions

This study investigated the thermophysical properties of two GFRP composites and a CFRP composite to provide data for engineering calculations and structural analyses. The research focused on the effects of cyclic thermal loading and high-temperature behaviour. Key findings are summarized below:

1. Thermogravimetric analysis-thermogravimetric (TG) analysis revealed qualitative similarities in mass change profiles for the two glass reinforced composites (GFRP A and GFRP B), with similar conventional onset temperatures (ONSET) of approximately 294 °C. However, distinct differences in weight change and a significantly higher ONSET temperature (approximately 355 °C) for the CFRP composite compared to the glass composites. Given the differences in ONSET TG and ONSET DTG temperatures, the lower DTG values of 230 °C for the GFRP composites and 340 °C for the CFRP composite should be used as a basis for establishing limits for subsequent measurements and defining operational limits.
2. Microcalorimetric analysis–(i) a glass transition in the CFRP composite, shifting from 95–110 °C (as-delivered) to 110–130 °C after heating to 280 °C; (ii) a strong likelihood of a similar glass transition in the glass-filled composite but with a difficult to determine transition temperature value, (iii) a slight decrease in specific heat for the CFRP composite after exposure to high temperatures.
3. Diffusivity studies-the CFRP composite exhibited the highest transverse and longitudinal thermal diffusivity. While the longitudinal diffusivity was approximately twice the transverse diffusivity for the glass composites, this ratio was closer to 10:1 for the graphite composite, consistent with previous findings [22]. Moreover, for carbon fiber/epoxy resin, a similar relationship-exhibiting higher longitudinal thermal diffusivity than transverse diffusivity-had also been reported previously, e.g., in [21,28]. The study investigates the thermal anisotropy of reinforced carbon fiber/epoxy composites, revealing that the obtained anisotropy ratio was even higher than in our research, primarily due to the enhanced in-plane thermal conductivity achieved through the introduction of high thermal conductive coaxial PAN/PBO carbon fibers.
4. The obtained results not only confirm the benefits of comprehensive testing but also illustrate the potential of using comparative analysis to cross-validate the obtained results. The measurement data from the studies of structures with imperfections provide an insight into the possible deviations of the effective property values from those obtained in the studies of well-defined structures.

## Figures and Tables

**Table 1:** DSC temperature program data.

Program	Temperature Range [*t*_min_; *t*_max_], °C	Rate of Temperature Change, K/min.	Number of Subintervals of the Range [*t*_min_; *t*_max_]	Number of Repetitions of Cycles
L	[−20; 120]	±5	4	2
M	[−20; 280]	±10	6	2.5

**Table 2:** Porosity and effective density of the structures studied.

Type of Reinforcement	Filling	Indication	Porosity, %	Density (Effective), kg/m^3^
glass	mat, layers	GFRP A	33.0	1407 ± 3.4
glass	mat, layers	GFRP B	3.5	1677 ± 1.4
graphite	fabric, layers	CFRP	0.2	1484 ± 0.9

**Table 3:** Calculated mass fraction of reinforcement in a solid structure phase based on image analysis.

Code	Volume Fraction of Reinforcement, %	Density of Reinforcement, kg/m^3^	Density of Matrix, kg/m^3^	Mass Fraction of Reinforcement, %
GFRP A	56 ± 7	2.40	1.2	72
GFRP B	43 ± 6	2.40	1.2	60
CFRP	57 ± 4	1.94	1.2	68

**Table 4:** Weighting data of samples subjected to TG test: initial weight, total weight change in test, final weight.

Structure	Sample Indication	*m*_post_, mg	∆*m*, mg	*m*_kon_, mg	Residue, % by Weight
GFRP A	specimen 1	7.61	−5.03	2.58	33.90
	specimen 2	12.45	−7.04	5.41	43.45
GFRP B	specimen 1	21.52	−10.74	10.78	50.09
	specimen 2	10.09	−5.03	5.06	50.15
CFRP	specimen 1	32.05	−10.86	21.19	66.12
	specimen 2	50.36	−18.43	31.93	63.40

**Table 5:** Coefficients of the DSC data fitting polynomial, i.e., data of the representative dependence of the specific heat of the GFRP B and CFRP structures on the temperature.

*i*	*μ_i_*; *n* = 3 [See Equation (2)]	
	GFRP B	CFRP
0	0.9770227	0.8444762
1	3.4229070 × 10^−3^	3.593296 × 10^−3^
2	5.8989400 × 10^−6^	−1.490891 × 10^−5^
3	−3.0474150 × 10^−8^	1.912467 × 10^−7^

**Table 6:** Coefficients of the transversal thermal diffusivity data fitting polynomial (see Equation (3); for GFRP A and GFRP B data linear fitting was applied).

*i*	*μ_i_*; *n* = 3 [See Equation (3)]		
	GFRP A	GFRP B	CFRP
0	0.1847094	0.2230017	0.2651952
1	−4.376068 × 10^−4^	−4.129915 × 10^−4^	2.678888 × 10^−4^
2	n.a.	n.a.	−1.297996 × 10^−7^
3	n.a.	n.a.	−9.988168 × 10^−9^

**Table 7:** Numerical data of longitudinal thermal diffusivity of the tested structures at RT.

*a*_in-plane_, mm^2^·s^−1^		
GFRP A	GFRP B	CFRP
0.323	0.498	2.628

**Table 8:** Calculated values of the effective thermal conductivity directional components of the investigated structures (data of the GFRP A structure determined using the specific heat value of the GFRP B structure are highlighted in italics).

	GFRP A	GFRP B	CFRP
*k_p_*_,eff,in-plane_, W·m^−1^·K^−1^	0.475	0.875	3.556
*k*_eff,out-of-plane_, W·m^−1^·K^−1^	0.259	0.378	1.234

**Table 9:** Literature comparative data on selected thermophysical parameters for RT.

	GFRP	CFRP	
	[23]	[22]	[21]
*ρ*, kg·m^−3^	1769	1468	1575
*c_p_*_,eff_, J·g^−1^·K^−1^	930	846	740
*a*_out-of-plane_, mm^2^·s^−1^	0.19	0.36	0.17
*a*_in-plane_, mm^2^·s^−1^	0.32	2.0	2.8

**Table 10:** Data and calculation results for determining the composition of composite structures based on the results of specific heat measurements for RT.

Structure	*c_p_*, J·g^−1^·K^−1^			Reinforcement Mass Fraction, %
	Matrix [24]	Filling [11,25]	Effective	
GFRP B	1.300	0.78	1.0476	48.5
CFRP	1.300	0.71	0.9119	65.8

## Data Availability

The original contributions presented in the study are included in the article, further inquiries can be directed to the corresponding author.

## Abbreviations

The following abbreviations are used in this manuscript:

CFRP	Carbon-fiber Reinforced Polymer
CTE	Coefficient of Thermal Expansion
DSC	Differential Scanning Calorimetry
GFRP	Glass-fiber Reinforced Polymer
LFA	Laser Flash Analysis
RT	Room Temperature
SEM	Scanning Electron Microscope
TG/DTG	Thermogravimetry/Differential Thermogravimetric
